# Assessment of Costs of Avoidable Delays in Intensive Care Unit Discharge

**DOI:** 10.1001/jamanetworkopen.2020.13913

**Published:** 2020-08-19

**Authors:** Sean M. Bagshaw, Dat T. Tran, Dawn Opgenorth, Xiaoming Wang, Danny J. Zuege, Armann Ingolfsson, Henry T. Stelfox, Nguyen X. Thanh

**Affiliations:** 1 Department of Critical Care Medicine, Faculty of Medicine and Dentistry, University of Alberta, Edmonton, Canada; 2 Critical Care Strategic Clinical Network, Alberta Health Services, Edmonton, Canada; 3 School of Public Health, University of Alberta, Edmonton, Canada; 4 Institute of Health Economics, Edmonton, Alberta, Canada; 5 Faculty of Pharmacy and Pharmaceutical Sciences, University of Alberta, Edmonton, Canada; 6 Health Services Statistical and Analytic Methods, Alberta Health Services, Edmonton, Canada; 7 Department of Critical Care Medicine, Cumming School of Medicine, University of Calgary, Alberta Health Services, Calgary, Canada; 8 Alberta School of Business, University of Alberta, Edmonton, Canada; 9 O’Brien Institute for Public Health, Cumming School of Medicine, University of Calgary, Calgary, Alberta, Canada; 10 Strategic Clinical Networks, Alberta Health Services, Edmonton, Canada

## Abstract

**Importance:**

Delays in transfer for discharge-ready patients from the intensive care unit (ICU) are increasingly described and contribute to strained capacity.

**Objective:**

To describe the epidemiological features and health care costs attributable to potentially avoidable delays in ICU discharge in a large integrated health care system.

**Design, Setting, and Participants:**

This population-based cohort study was performed in 17 adult ICUs in Alberta, Canada, from June 19, 2012, to December 31, 2016. Participants were patients 15 years or older admitted to a study ICU during the study period. Data were analyzed from October 19, 2018, to May 20, 2020.

**Exposures:**

Avoidable time in the ICU, defined as the portion of total ICU patient-days accounted for by avoidable delay in ICU discharge (eg, waiting for a ward bed).

**Main Outcomes and Measures:**

The primary outcome was health care costs attributable to avoidable time in the ICU. Secondary outcomes were factors associated with avoidable time, in-hospital mortality, and measures of use of health care resources, including the number of hours in the ICU and the number of days of hospitalization. Multilevel mixed multivariable regression was used to assess associations between avoidable time and outcomes.

**Results:**

In total, 28 904 patients (mean [SD] age, 58.3 [16.8] years; 18 030 male [62.4%]) were included. Of these, 19 964 patients (69.1%) had avoidable time during their ICU admission. The median avoidable time per patient was 7.2 (interquartile range, 2.4-27.7) hours. In multivariable analysis, male sex (odds ratio [OR], 0.92; 95% CI, 0.87-0.98), comorbid hemiplegia or paraplegia (OR 1.47; 95% CI, 1.23-1.75), liver disease (OR 1.20; 95% CI, 1.04-1.37), admission Acute Physiology and Chronic Health Evaluation II score (OR, 1.03; 95% CI, 1.02-1.03), surgical status (OR, 0.90; 95% CI, 0.82-0.98), medium community hospital type (OR, 0.12; 95% CI, 0.04-0.32), and admission year (OR, 1.16; 95% CI, 1.13-1.19) were associated with avoidable time. The cumulative avoidable time was 19 373.9 days, with estimated attributable costs of CAD$34 323 522. Avoidable time accounted for 12.8% of total ICU bed-days and 6.4% of total ICU costs. Patients with avoidable time before ICU discharge showed higher unadjusted in-hospital mortality (1115 [5.6%] vs 392 [4.4%]; *P* < .001); however, in multivariable analysis, avoidable time was associated with reduced in-hospital mortality (adjusted hazard ratio, 0.74; 95% CI, 0.64-0.85). Results were similar in sensitivity analyses.

**Conclusions and Relevance:**

In this study, potentially avoidable discharge delay occurred for most patients admitted to ICUs across a large integrated health system and translated into substantial associated health care costs.

## Key Points

**Question:**

What is the association between avoidable intensive care unit (ICU) discharge delay and health care costs and patient outcomes?

**Findings:**

In this cohort study of 28 904 adult patients treated in the ICU, delayed discharge occurred in 19 964 (69.1%). This avoidable time in the intensive care unit accounted for 12.8% of total ICU bed-days and 6.4% of total ICU costs.

**Meaning:**

Avoidable discharge delays occurred in most patients in this study, incurring substantial health care costs; strategies at mitigation of potentially avoidable time in the ICU could realize improved efficiency and costs savings for the health care system.

## Teaser

This population-based cohort study describes the epidemiology and health care costs associated with potentially avoidable delays in intensive care unit discharge among adult patients in a large integrated health care system.

## Introduction

Transfer from the intensive care unit (ICU) to the ward generally coincides with a patient’s recovery from critical illness and planned stepdown to lower-intensity care. Timely discharge from ICU is important to preserve capacity, facilitate patient flow, and ensure bed availability for additional critically ill patients in need of advanced organ support.

Delay in transfer from ICU is commonly described and may contribute to strained capacity.^1,2^ Prior work, generally from small studies during short periods, has suggested that 20% to 50% of patients experience delay in ICU discharge, most often attributed to a lack of ward bed availability.^1,3^ In these studies, discharge delay was variably defined and showed inconsistent associations with risk for patient adverse events (eg, disrupted sleep, delirium, nosocomial infection) and outcomes (eg, mortality), although discharge delay generally portended greater resource use (eg, prolonged hospital stay)^4^ and costs.^5,6^ Discharge delay may also be associated with unplanned after-hours discharge or direct discharge home from the ICU, a care process most ICUs are not traditionally prepared to manage.^1,7,8^

Discharge delay has been recognized as a key indicator of ICU quality and hospital performance.^9^ *Avoidable time* in the ICU has been defined as the portion of a total ICU stay accounted for by avoidable delay in ICU discharge.^10^ The aim of our study was to describe the population-based incidence of avoidable time in the ICU and evaluate the association between avoidable time and health care costs and patient outcomes. We hypothesized that delays in ICU discharge would be common and would be associated with adverse outcomes and excess costs to the health care system.

## Methods

This study was approved by the Research Ethics Board at the University of Alberta, Edmonton, Canada, which waived the need for written informed consent for the use of prospectively collected data. This study followed the Strengthening the Reporting of Observational Studies in Epidemiology (STROBE) reporting guideline.

### Study Design, Population, and Setting

This was a population-based cohort study of all patients 15 years or older admitted to all 17 adult ICUs in 14 hospitals (14 mixed medical/surgical units, 2 cardiovascular surgical ICUs, and 1 neurosciences ICU) across 7 cities in Alberta, Canada, from June 19, 2012, to December 31, 2016 (eTable 1 in the Supplement).^10^ For patients with multiple ICU admissions during the index hospitalization, only the first ICU admission was analyzed. Patients who died in the ICU were excluded. In Alberta (2016 population, approximately 4.1 million),^11^ health services are predominantly administered with a single provincial provider, Alberta Health Services.^12^ Alberta has an ICU bed base of approximately 9.8 ICU beds per 100 000 population (compared with 12.9 ICU beds per 100 000 population nationwide in Canada and 20 ICU beds per 100 000 population in the United States)^13,14,15^ and is perceived to routinely operate under conditions of strained ICU capacity.^16^

### Data Sources

Data were obtained from an ICU-specific clinical information system/data repository (eCritical/TRACER) and Alberta administrative health data sets, including the Discharge Abstract Database.^17^ eCritical is composed of a bedside system (eCritical MetaVision; iMDsoft), which provides electronic interdisciplinary clinical documentation and collation of demographic, diagnostic/case-mix, acuity (Acute Physiology and Chronic Health Evaluation [APACHE] II score), laboratory, and device data. eCritical TRACER provides a comprehensive, multimodal, integrated data repository of patient-specific ICU data enabling creation of reports and data extracts for administrative, quality, and research purposes. Data within eCritical systems undergo rigorous data quality assurance and audit.^18^ eCritical/TRACER has routinely supported health services research.^7,19,20,21^

The Discharge Abstract Database provides rich data on all hospitalizations in Alberta.^17^ It contains patient demographic data, interventions/procedures, lengths of stay, discharge disposition, most responsible diagnoses, and comorbidities.^22,23^

### Primary Exposure

The primary exposure was avoidable time, defined as the portion of total ICU stay accounted for by avoidable delay in ICU discharge. In Alberta, ICU avoidable discharge delay has been defined as the time difference between the date/time of decision for transfer and when the patient was actually discharged from the ICU, minus 4 hours.^10^ Site-specific avoidable time is captured and reported as an ICU key performance indicator in Alberta.

### Outcomes

The primary outcome was health care costs attributable to avoidable time. Secondary outcomes evaluated factors associated with avoidable time, in-hospital mortality, and measures of use of health care resources, including hours in ICU and days of hospitalization.

### Statistical Analysis

Data were analyzed from October 19, 2018, to May 20, 2020. Clinical and demographic characteristics of patients and characteristics of ICUs were described and compared between patients having and not having avoidable time (measured in days) using univariable and multivariable analyses. For the univariable analysis, χ^2^ and 2-tailed *t* tests were used for categorical and continuous variables, respectively. For the multivariable analysis, we used logistic regression to evaluate factors associated with avoidable time (yes or no). Independent variables were clinical and sociodemographic characteristics of patients, including age, sex, means of median household income at the forward sortation area level,^24^ comorbidities,^23^ primary diagnostic system, admission ICU category, surgical status, admission APACHE II score, admission time, bed occupancy at time of admission, year of admission, and type of hospital. To account for potential random effects among different ICUs, we used a multilevel mixed-effects regression (patient and ICU levels). Of note, we used a likelihood ratio test to examine inclusion of potential risk adjustment factors; that is, except for the primary variables (age, sex, and year of admission), a variable remained in the final model if the likelihood ratio test was significant at a 5% level.

Health care costs of avoidable time were estimated by a modeling technique. The total costs of avoidable time were a sum of the costs of avoidable time (in days) of patients in the ICU who were discharged to the ward and the costs of avoidable time (in days) of those who were discharged home. The costs of avoidable time among ward-discharged patients were estimated by multiplying the difference in costs per day between the ICU and ward with the avoidable time (in days) of ward-discharged patients. The costs of avoidable time of home-discharged patients were estimated by multiplying the difference in costs per day between the ICU and home with the number of avoidable days of home-discharged patients. We used the ICU cost per day at CAD$3545 (US $2642) and the ward cost per day at CAD$2079 (US $1549), as previously estimated.^10^ The home cost per day was assumed to be CAD$0; however, we performed sensitivity analysis on overall costs if home costs per day ranged from 0 to 100% of ward discharge costs. For this cost analysis, we calculated costs of avoidable time per year in Alberta and performed sensitivity analyses for the hypothetical cost savings and added ICU-bed capacity per year if avoidable time was reduced by 5%, 10%, 25%, and 50%, based on the total costs of avoidable time in 2016. All costs were converted to 2019 Canadian dollars using the Consumer Price Index published by Statistics Canada (eTable 2 in the Supplement).

For the association between avoidable time and in-hospital mortality, we used 3 multilevel mixed-effects Cox regressions with the time from ICU discharge to in-hospital death (or hospital discharge for survivors) as the outcome variable (0 or 1) and avoidable time as the main independent variable. In the first regression, avoidable time was treated as a binary variable (having or not having any avoidable time). In the second regression, number of avoidable days was treated as a continuous variable. Finally, in the third regression, avoidable time was treated as a categorical variable (eg, 0 days, 1-3 days, ≥4 days, etc) to examine whether there was a gradient or U-shaped association. Covariates in these 3 regressions were the same as described above. STATA MP, version 13.0 (StataCorp LLC), was used for analyses. Two-sided *P* < .05 indicated significance.

## Results

**Figure 1.**
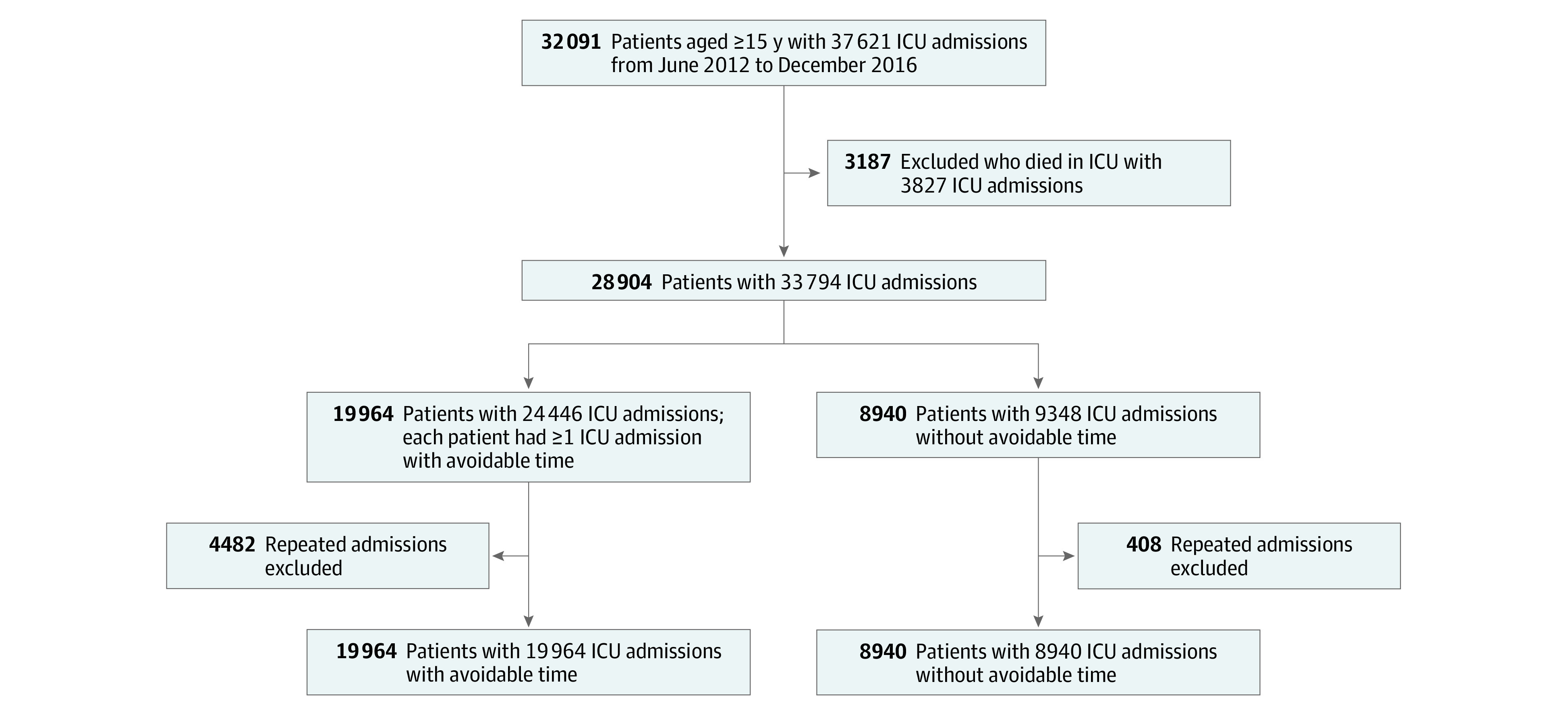
Patient Selection Flowchart ICU indicates intensive care unit.

In total, there were 37 621 ICU admissions for 32 091 unique patients 15 years or older from June 19, 2012, to December 31, 2016 (Figure 1). After excluding 3187 patients who died in the ICU (10.2%; of whom 185 [5.8%] had avoidable time) and 1918 repeated ICU admissions, we included 28 904 patients for analysis (mean [SD] age, 58.3 [16.8] years; 18 030 male [62.4%] and 10 874 female [37.6%]). Of these, 19 964 patients (69.1%) had avoidable time and 8940 (30.9%) had no avoidable time during their ICU admission. The proportion of patients with avoidable time showed variation across ICUs, ranging from 25.3% to 82.3% (eFigure 1 in the Supplement).

The mean (SD) avoidable time per patient was 23.3 (34.3) hours; the median, 7.2 (interquartile range [IQR], 2.4-27.7) hours (Table 1). In multivariable analysis, factors associated with avoidable time included male sex (odds ratio [OR], 0.92; 95% CI, 0.87-0.98); comorbid hemiplegia or paraplegia (OR, 1.47; 95% CI, 1.23-1.75), liver disease (OR, 1.20; 95% CI, 1.04-1.37), chronic pulmonary disease (OR, 1.16; 95% CI, 1.08-1.25), and peptic ulcer disease (OR, 1.18; 1.02-1.38); admission related to primary respiratory (OR, 1.20; 95% CI, 1.08-1.34), gastrointestinal tract (OR, 1.34; 95% CI, 1.18-1.52), or transplant (OR, 1.81; 95% CI, 1.33-2.45) diagnosis; surgical status (OR, 0.90; 95% CI, 0.82-0.98); admission APACHE II score (OR, 1.03; 95% CI, 1.02-1.03); medium community hospital type (OR, 0.12; 95% CI, 0.04-0.32); and admission year (OR, 1.16; 95% CI, 1.13-1.19) (Table 2).

**Table 1.** Basic Characteristics of the Study Cohort Stratified by Avoidable Time

Variable	Study group^a^			*P* value
	All (N = 28 904)	Any avoidable time (n = 19 964)	No avoidable time (n = 8940)	
Female	10 874 (37.6)	7786 (39.0)	3088 (34.5)	<.001
Age, mean (SD), y	58.3 (16.8)	58.3 (16.8)	58.4 (16.8)	.48
Household income, CAD$				
0 to 40 000	479 (1.7)	363 (1.8)	116 (1.3)	<.001
>40 000 to 60 000	4399 (15.2)	3114 (15.6)	1285 (14.4)	
>60 000 to 80 000	13 880 (48.0)	9577 (48.0)	4303 (48.1)	
>80 000 to 100 000	6072 (21.0)	4171 (20.9)	1901 (21.3)	
>100 000	4074 (14.1)	2739 (13.7)	1335 (14.9)	
Selected comorbidities^b^				
Myocardial infarction	4139 (14.3)	2556 (12.8)	1583 (17.7)	<.001
Congestive heart failure	4785 (16.6)	3262 (16.3)	1523 (17.0)	.14
Peripheral vascular disease	2475 (8.6)	1643 (8.2)	832 (9.3)	.002
Cerebrovascular disease	2704 (9.4)	1910 (9.6)	794 (8.9)	.06
Dementia	484 (1.7)	374 (1.9)	110 (1.2)	<.001
Chronic pulmonary disease	5576 (19.3)	4150 (20.8)	1426 (16.0)	<.001
Rheumatic disease	632 (2.2)	476 (2.4)	156 (1.7)	.001
Diabetes	8148 (28.2)	5682 (28.5)	2466 (27.6)	.13
Hemiplegia or paraplegia	856 (3.0)	677 (3.4)	179 (2.0)	<.001
Cancer	3429 (11.9)	2604 (13.0)	825 (9.2)	<.001
Peptic ulcer	1125 (3.9)	878 (4.4)	247 (2.8)	<.001
Chronic kidney disease	2296 (7.9)	1728 (8.7)	568 (6.4)	<.001
Mild liver disease	1603 (5.5)	1296 (6.5)	307 (3.4)	<.001
Moderate or severe liver disease	1001 (3.5)	832 (4.2)	169 (1.9)	<.001
Metastatic solid tumor	1438 (5.0)	1121 (5.6)	317 (3.5)	<.001
Charlson comorbidity index, mean (SD)^c^	2.3 (2.5)	2.4 (2.6)	2.0 (2.2)	<.001
0	7931 (27.4)	5249 (26.3)	2682 (30.0)	<.001
1	6276 (21.7)	4152 (20.8)	2124 (23.8)	
2	4027 (13.9)	2800 (14.0)	1227 (13.7)	
>2	10 670 (36.9)	7763 (38.9)	2907 (32.5)	
Primary diagnostic system				
Cardiovascular	10 700 (37.0)	6252 (31.3)	4448 (49.8)	<.001
Gastrointestinal tract	3141 (10.9)	2481 (12.4)	660 (7.4)	
Genitourinary	890 (3.1)	636 (3.2)	254 (2.8)	
Hematology	57 (0.2)	45 (0.2)	12 (0.1)	
Metabolic/endocrine	578 (2.0)	428 (2.1)	150 (1.7)	
Musculoskeletal/skin	793 (2.7)	584 (2.9)	209 (2.3)	
Neurological	4533 (15.7)	3174 (15.9)	1359 (15.2)	
Respiratory	5535 (19.1)	4310 (21.6)	1225 (13.7)	
Transplant	331 (1.1)	273 (1.4)	58 (0.6)	
Trauma	2072 (7.2)	1607 (8.0)	465 (5.2)	
Other	274 (0.9)	174 (0.9)	100 (1.1)	
Admission ICU category				
Medical	11 316 (39.2)	8573 (42.9)	2743 (30.7)	<.001
Neurological	2172 (7.5)	1580 (7.9)	592 (6.6)	
Surgical	13 747 (47.6)	8514 (42.6)	5233 (58.5)	
Trauma	1669 (5.8)	1297 (6.5)	372 (4.2)	
Surgery				
Elective	9535 (33.0)	5596 (28.0)	3939 (44.1)	<.001
Emergency	4999 (17.3)	3488 (17.5)	1511 (16.9)	
Nonoperative	14 370 (49.7)	10 880 (54.5)	3490 (39.0)	
Admission APACHE II score, mean (SD)	17.3 (7.1)	18.0 (7.2)	15.8 (6.6)	<.001
After-hours admission	7441 (25.7)	5544 (27.8)	1897 (21.2)	<.001
In-hospital mortality	1507 (5.2)	1115 (5.6)	392 (4.4)	<.001
Hospital type				
Teaching	25 772 (89.2)	17 872 (89.5)	7900 (88.4)	<.001
Large community	2982 (10.3)	2054 (10.3)	928 (10.4)	
Medium community	150 (0.5)	38 (0.2)	112 (1.3)	
Admission year				
2012	1150 (4.0)	750 (3.8)	400 (4.5)	<.001
2013	4406 (15.2)	2821 (14.1)	1585 (17.7)	
2014	7361 (25.5)	5217 (26.1)	2144 (24.0)	
2015	7356 (25.4)	5047 (25.3)	2309 (25.8)	
2016	8631 (29.9)	6129 (30.7)	2502 (28.0)	
Avoidable time, h^d^				
Mean (SD)	NA	23.3 (34.3)	NA	NA
Median (IQR)	NA	7.2 (2.4-27.7)	NA	NA

Abbreviations: APACHE, Acute Physiologic and Chronic Health Evaluation; ICU, intensive care unit; IQR, interquartile range; NA, not applicable.

^a^ Unless otherwise indicated, data are expressed as number (percentage) of patients. Percentages have been rounded and may not total 100.

^b^ Patients may have more than 1 comorbidity.

^c^ Higher scores indicate greater number of comorbidities.

^d^ Defined as the proportion of total ICU stay accounted for by avoidable delay in ICU discharge (calculated in hours). ICU avoidable discharge delay is defined as the time difference between the date and time of decision for transfer and when the patient was actually discharged from the ICU minus 4 hours.

**Table 2.** Factors Associated With Having Avoidable Time^a^

Variable	OR (95% CI)	
	Univariable analysis	Multivariable analysis
Sex		
Female	1 [Reference]	1 [Reference]
Male	0.93 (0.88-0.98)	0.92 (0.87-0.98)
Age, y		
18-64	1 [Reference]	1 [Reference]
65-74	1.13 (1.06-1.20)	0.99 (0.92-1.06)
75-84	1.17 (1.08-1.26)	1.01 (0.93-1.10)
≥85	1.07 (0.90-1.26)	0.93 (0.79-1.11)
Comorbid disease		
Hemiplegia or paraplegia	1.48 (1.24-1.76)	1.47 (1.23-1.75)
Chronic pulmonary disease	1.25 (1.17-1.34)	1.16 (1.08-1.25)
Mild liver disease	1.40 (1.23-1.60)	1.20 (1.04-1.37)
Peptic ulcer disease	1.34 (1.16-1.56)	1.18 (1.02-1.38)
Primary diagnostic system		
Cardiovascular	1 [Reference]	1 [Reference]
Gastrointestinal tract	1.27 (1.23-1.44)	1.34 (1.18-1.52)
Genitourinary	0.81 (0.69-0.97)	0.86 (0.72-1.03)
Hematology	1.33 (0.69-2.58)	1.49 (0.77-2.89)
Metabolic/endocrine	1.14 (0.92-1.42)	1.23 (0.99-1.53)
Musculoskeletal/skin	0.87 (0.72-1.04)	1.00 (0.83-1.21)
Neurological	0.86 (0.77-0.97)	0.90 (0.80-1.01)
Respiratory	1.16 (1.04-1.29)	1.20 (1.08-1.34)
Transplant	1.91 (1.42-2.58)	1.81 (1.33-2.45)
Trauma	0.95 (0.82-1.09)	1.15 (0.92-1.42)
Other	0.87 (0.67-1.13)	1.02 (0.79-1.33)
Admission ICU category		
Medical	1 [Reference]	1 [Reference]
Neurological	0.97 (0.85-1.10)	1.14 (0.99-1.32)
Surgical	0.84 (0.78-0.91)	0.90 (0.82-0.98)
Trauma	0.84 (0.74-0.97)	0.97 (0.77-1.22)
Admission APACHE II score	1.03 (1.03-1.04)	1.03 (1.02-1.03)
After-hours admission	0.92 (0.86-0.99)	0.89 (0.83-0.96)
Hospital type		
Teaching	1 [Reference]	1 [Reference]
Large community	0.87 (0.54-1.41)	0.72 (0.46-1.15)
Medium community	0.14 (0.05-0.39)	0.12 (0.04-0.32)
Admission year	1.14 (1.12-1.17)	1.16 (1.13-1.19)

Abbreviations: APACHE, Acute Physiologic and Chronic Health Evaluation; ICU, intensive care unit; OR, odds ratio.

^a^ Determined as yes or no (n = 28 904).

**Figure 2.**
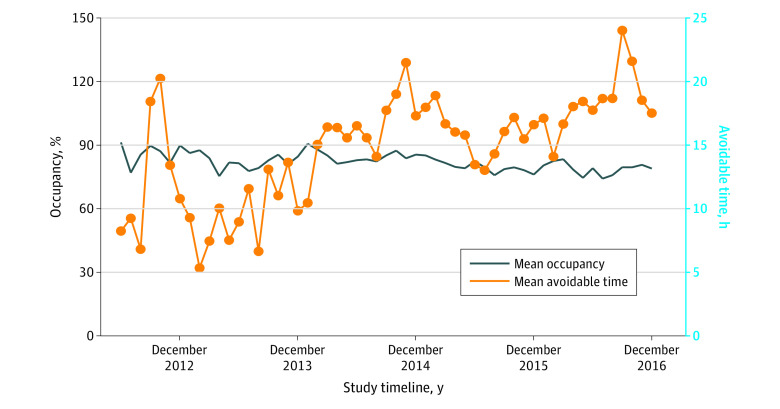
Monthly Mean Avoidable Time During the Study Period

During the study period, an increase in avoidable time occurred, despite stability in mean ICU occupancy (adjusted OR per year, 1.08; 95% CI, 1.05-1.10) (Figure 2). The proportion of ICU admissions with avoidable time also showed an increasing trend during the study (Table 3). In total, 2860 patients were discharged directly home from the ICU (9.9%), of whom 1656 (57.9%) had avoidable time (median, 24.8 [IQR, 6.8-53.0] hours), compared with 26 044 patients who were discharged to the ward (90.1%), of whom 18 309 (70.3%) had avoidable time (median, 6.2 [IQR, 2.3-26.8] hours). The proportion of patients discharged directly home increased during the study (adjusted OR per year, 1.09; 95% CI, 1.04-1.14).

**Table 3.** Amount and Cost of Avoidable Time by Type of Discharge Location and Year

Year^a^	Avoidable time, d			Estimated cost of avoidable time, CAD$^b^		
	Ward discharge	Home discharge	Total	Ward discharge	Home discharge	Total
2012	608.8	79.7	688.5	892 479	282 513	1 174 992
2013	1700.3	222.5	1922.8	2 492 606	788 823	3 281 429
2014	4401.8	713.7	5115.5	6 453 042	2 530 188	8 983 230
2015	4259.7	730.1	4989.7	6 244 652	2 588 182	8 832 834
2016	5555.2	1102.2	6657.3	8 143 912	3 907 125	12 051 037
Total	16 525.7	2848.2	19 373.9	24 226 691	10 096 831	34 323 522

^a^ Data were collected from June 19, 2012, to December 31, 2016.

^b^ To convert to US $, multiply by 0.75.

During the study period, the cumulative avoidable time in the ICU was 19 373.9 days, with 16 252.7 days for patients discharged to the ward and 2848.2 days for patients discharged directly home (Table 3 and eFigure 2 in the Supplement). This avoidable time translated into 12.8% of total ICU days (range, 8.3%-15.4% annually) (eTable 3 in the Supplement).

The estimated total costs attributed to avoidable delays in ICU transfer was CAD$34 323 522 (US $25 581 160; approximately CAD$9.96 million [US $7.42 million] per year during the last 3 years of the study), with CAD$24 226 691 (US $18 056 039) for those discharged to the ward and CAD$10 096 831 (US $7 525 121) for those directly discharged home. This avoidable time was estimated to be 6.4% of total ICU costs (range, 4.0%-7.8% annually), calculated as the costs of the proportion of avoidable days to total ICU-days (eTable 4 in the Supplement). In sensitivity analysis, when the costs of home discharge were assumed to be proportional to the costs of a ward discharge day (0%, 25%, 50%, and 100%), the total ICU costs attributed to avoidable time ranged from 4.4% to 6.4% (eTable 5 in the Supplement).

In sensitivity analysis, if avoidable time was reduced by 5%, 10%, 25%, and 50%, the range in potential cost savings would be CAD$0.60 to CAD$6.03 million (US$ 0.45 to US $4.49 million) per year (eTable 6 in the Supplement). This would correspond to additional ICU-bed capacity per year ranging from 0.47 beds for a 5% reduction to 4.66 beds for a 50% reduction in avoidable time.

Patients with avoidable time showed higher unadjusted in-hospital mortality (1115 [5.6%] vs 392 [4.4%]; *P* < .001); however, in multivariable analysis, avoidable time was associated with reduced in-hospital mortality (adjusted hazard ratio [HR], 0.74; 95% CI, 0.64-0.85) (eTable 7 in the Supplement). The results were similar when avoidable time was modeled as a continuous variable (adjusted HR per 1-hour increase in avoidable time, 0.998; 95% CI, 0.996-0.999) and when further categorized by duration (eTable 8 in the Supplement). Mean (SD) duration of ICU stay (142.4 [192.7] vs 87.1 [138.2] hours; mean difference, −55.3 [95% CI, −59.7 to −50.9] hours), total hospital stay (25.4 [41.6] vs 16.4 [29.2] days; mean difference, −8.9 [95% CI, −9.9 to −8.0] days), ICU stay before readiness for discharge (115 [186] vs 83 [138] hours; mean difference, −31.9 [95% CI, −36.3 to −27.7] hours), and duration of post-ICU hospital stay (16.9 [35.7] vs 10.3 [24.9] days; mean difference, −6.6 [95% CI, −7.4 to −5.8] days) were all longer for patients with avoidable time compared with patients with no avoidable time (eTable 9 in the Supplement).

## Discussion

In this multicenter population-based cohort study, potentially avoidable delays in ICU discharge occurred in approximately 7 of 10 patients, and, although not translating into incremental mortality risk, avoidable time was directly associated with substantial health care costs. The median ICU discharge delay was approximately 7 hours; however, the delay exceeded 24 hours for 1 in 4 patients. During the study, temporal trends imply increasing occurrence of avoidable time and direct discharges to home across ICUs. Several patient- and hospital-level factors were associated with avoidable time, including female sex, selected comorbidities, higher illness acuity, nonsurgical diagnosis, and admission to a teaching hospital. Avoidable time represented CAD$34 million (US $25 million) in added expenses and accounted for 12.8% of all ICU days and 6.4% of total ICU health care costs. A reduction in avoidable time by 25% to 50% would equate to the estimated operational costs of adding 2 to 5 ICU beds per year. Although complete recovery of these costs would be implausible, these observations provide important new knowledge on the potential opportunity costs of mitigating avoidable delays in ICU discharge.

### Context With Prior Literature

Few studies have rigorously described the epidemiology, outcomes, and attributable health care costs associated with ICU discharge delay.^1,3,4,5,6^ Prior work has notable limitations and lacks generalizability owing to being relatively small (ie, performed in only 1 or relatively few ICUs), being restricted to academic/tertiary centers focusing on either surgical or medical ICUs, and using heterogeneous definitions for discharge delay (ie, ranging from 6 to 24 hours).^1,2,4,5,6^ For example, in a single surgical ICU,^5^ 22% of patients experienced discharge delay, defined as occurring more than 24 hours after discharge readiness. Similarly, a retrospective study in 2 surgical ICUs^6^ found discharge delay occurred in 24.8% of patients, defined as occurring beyond 2:30 pm for discharge decisions made before 9:00 am. In another prospective cohort study performed during 3 months in 5 mixed ICUs,^4^ 49.9% of patients had discharge delay, defined as occurring more than 6 hours following planned discharge. Finally, in a single tertiary-care mixed ICU for 16 months,^2^ 18% of patients had ICU discharge delay, defined as failure to transfer within 24 hours. Our data support and extend these findings by showing that most patients experience discharge delays from the ICU and that these delays often exceed more than 1 day.

The most common reason for ICU discharge delay is a lack of an available ward bed, which was shown in prior work to range from 46% to 92%.^2,4,5,6^ This issue can be exacerbated in institutions with a high prevalence of patients with antimicrobial-resistant organisms, whereby isolation precautions are mandated on the ward.^5^ In 1 study,^2^ 67% of discharges were delayed owing to unavailability of ward beds and/or concerns from the transfer service; whereas in 33% of patients, the delay was actually due to clinical deterioration before transfer. Although our study was not able to specifically explain the nuanced reasons for discharge delay, the observations from other studies suggest that the issue of ICU discharge delay is predominantly attributed to hospital-level organization and is directly affected by global hospital census. We submit that such delays can impede the timely management of ICU capacity and patient flow.^5^

Our study, along with prior work,^4^ shows that patients experiencing ICU discharge delay had longer total durations of stay in ICU and hospital. These potential avoidable delays in ICU discharge have substantial resource and cost implications.^5,6^ In 1 prospective cohort study,^6^ 2 surgical ICUs had approximately 185 avoidable days and €199 268 in attributable costs due to ICU discharge delay during 1 year. Similarly, in another single surgical ICU study,^5^ the attributable costs of discharge delays were estimated at US $21 547 per week (US $581 790 annually). We submit that our data extend these observations by showing that the attributable costs of potentially avoidable time approach 6.4% of total ICU health care costs (approximately CAD$34 million over 3 years) across an entire provincial health system.

### Implications for Health Policy and Research

Avoidable time is a simple indicator of strained ICU capacity and should be measured, reported, and benchmarked as a key quality indicator for ICU and hospital performance.^9^ The observed increased trend in avoidable time has translated into organizational changes that directly affect patient care, such as after-hours discharge and direct hospital discharge from the ICU.^7,8,25,26^ In our study, 9.9% of patients were directly discharged home with an increasing trend evident during the study period, a process that was likely unplanned and often in response to lack of ward bed availability.^7^ We submit that this may present opportunities to redesign and implement innovative care models, such as pathways to facilitate direct discharge to the community for carefully selected patients or to transition to temporary step-down units to enable timely discharge of suitable patients and preserve ICU capacity.^27^

Although 1 reason to explain the burden of avoidable time in Alberta could relate to comparably fewer ICU beds per 100 000 population than other jurisdictions in Canada, an alternative explanation could relate to broader inefficiencies in use of acute care beds.^28^ One inference would be that costs savings could be realized through strategic reductions in ICU beds with redistribution of investments to creating lower-cost ward beds, particularly among ICUs with a high prevalence of avoidable time that is driven predominantly by lack of ward bed availability.^10^ Whether such reductions in ICU beds would translate into greater risk for adverse events during periods of genuine strain and limited ICU bed availability is uncertain and deserves further investigation.

### Limitations

This study has limitations that warrant consideration. First, although our study was large and multicentric and provides population-based estimates of avoidable time, we were unable to ascertain the specific reasons for ICU discharge delay. We also recognize that the association between avoidable time and mortality is susceptible to residual confounding. Second, we are unable to comment on whether upstream queueing occurred as a result of discharge-ready patients experiencing avoidable delays in transfer. Third, we excluded patients who died in the ICU. Although a small proportion (5.8%) had avoidable time before death, this did not substantially influence the cumulative avoidable time or attributable costs. Moreover, we were unable to ascertain whether such deaths were due to clinical deterioration while awaiting ICU discharge or were expected and aligned with the patient goals of care. Fourth, we applied a standard cost-per-day for both ICU and ward beds over time and recognize, particularly in the ICU, that costs may decrease concomitant with intensity of care. Although a large proportion of ICU bed-day costs are likely fixed, this standard may result in an inflated estimate of the costs attributable to avoidable time. We also recognize that patients experiencing avoidable time may have other reasons for added costs not attributable to avoidable time that are not captured in our study. Finally, the cost inputs for our analysis may not translate to other jurisdictions and may be sensitive to change over time. As such, we recognize that this study represents the perspective of an integrated provincial health region in Canada and may not be generalizable to other jurisdictions, although we submit the issue of potentially avoidable discharge delay and attributable costs are likely commonly encountered in contemporaneous ICU practice.^2,4,5,6^

## Conclusions

In this cohort study, potentially avoidable discharge delay occurred in 7 of 10 patients admitted to ICUs across a large integrated health region, with 1 of 4 patients having discharge delays exceeding 24 hours and avoidable time showing rising trends. Such avoidable time due to discharge delay adds substantial health care costs, and strategies at mitigation could realize both innovation in health services delivery and cost savings.

## Notes

Supplement. **eTable 1.** List of Participating ICUs **eTable 2.** Summary of Cost-Saving Analysis **eTable 3.** Avoidable Time in Days and as a Percentage of Total ICU Days by Year **eTable 4.** Costs of Avoidable Time in and as a Percentage of Total ICU Costs by Year **eTable 5.** Sensitivity Analysis of Increasing Home Cost Relative Ward Discharge Cost (per Day) on Total Health Care Costs Attributed to Avoidable Days **eTable 6.** Sensitivity Analysis of the Impact of Potential Reductions in Avoidable Time on Health Care Cost Savings per Year and Potential Added ICU-Bed Capacity That Would Be Available **eTable 7.** Mixed-Effects Cox Regression Model for Association Between In-Hospital Mortality and Having Avoidable-Time **eTable 8.** Strata of Avoidable-Time and Association With In-Hospital Mortality **eTable 9.** Summary of Duration of Stay Before ICU, Before Discharge Ready in ICU, and Total Lengths of Stay Stratified by Patients Who Did and Did Not Experience Avoidable Time **eFigure 1.** Summary of Distribution of Avoidable-Time Across Study ICUs During the Study Period **eFigure 2.** Summary of ICU Discharge Time Stratified by Avoidable-Time
