# Supplementary material for: Assessment of Costs of Avoidable Delays in Intensive Care Unit Discharge
Source: JAMA Netw Open. 2020 Aug 19;3(8):e2013913. doi: 10.1001/jamanetworkopen.2020.13913 (PMC7439109; doi:10.1001/jamanetworkopen.2020.13913)
Supplement: Supplement. — eTable 1. List of Participating ICUs eTable 2. Summary of Cost-Saving Analysis eTable 3. Avoidable Time in Days and as a Percentage of Total ICU Days by Year eTable 4. Costs of Avoidable Time in and as a Percentage of Total ICU Costs by Year eTable 5. Sensitivity Analysis of Increasing Home Cost Relative Ward Discharge Cost (per Day) on Total Health Care Costs Attributed to Avoidable Days eTable 6. Sensitivity Analysis of the Impact of Potential Reductions in Avoidable Time on Health Care Cost Savings per Year and Potential Added ICU-Bed Capacity That Would Be Available eTable 7. Mixed-Effects Cox Regression Model for Association Between In-Hospital Mortality and Having Avoidable-Time eTable 8. Strata of Avoidable-Time and Association With In-Hospital Mortality eTable 9. Summary of Duration of Stay Before ICU, Before Discharge Ready in ICU, and Total Lengths of Stay Stratified by Patients Who Did and Did Not Experience Avoidable Time eFigure 1. Summary of Distribution of Avoidable-Time Across Study ICUs During the Study Period eFigure 2. Summary of ICU Discharge Time Stratified by Avoidable-Time [file jamanetwopen-3-e2013913-s001.pdf]

Supplementary Online Content

Bagshaw SM, Tran DT, Opgenorth D, et al. Assessment of costs of avoidable delays in intensive care unit discharge. *JAMA Netw Open*. 2020;3(8):e2013913. doi:10.1001/jamanetworkopen.2020.13913

eTable 1. List of Participating ICUs

eTable 2. Summary of Cost-Saving Analysis

eTable 3. Avoidable Time in Days and as a Percentage of Total ICU Days by Year

eTable 4. Costs of Avoidable Time in and as a Percentage of Total ICU Costs by Year

eTable 5. Sensitivity Analysis of Increasing Home Cost Relative Ward Discharge Cost (per Day) on Total Health Care Costs Attributed to Avoidable Days

eTable 6. Sensitivity Analysis of the Impact of Potential Reductions in Avoidable Time on Health Care Cost Savings per Year and Potential Added ICU-Bed Capacity That Would Be Available

eTable 7. Mixed-Effects Cox Regression Model for Association Between In-Hospital Mortality and Having Avoidable-Time

eTable 8. Strata of Avoidable-Time and Association With In-Hospital Mortality

eTable 9. Summary of Duration of Stay Before ICU, Before Discharge Ready in ICU, and Total Lengths of Stay Stratified by Patients Who Did and Did Not Experience Avoidable Time

eFigure 1. Summary of Distribution of Avoidable-Time Across Study ICUs During the Study Period

eFigure 2. Summary of ICU Discharge Time Stratified by Avoidable-Time

This supplementary material has been provided by the authors to give readers additional information about their work.

©2020 Bagshaw SM et al. *JAMA Network Open*.

eTable 1. List of Participating ICUs

ICU name	Location	ICU type	Hospital type	Hospital classification (CIHI)*	eCritical implementation date
Foothills Medical Center Multi-Systems ICU	Calgary	Mixed (medical, surgical, neurosurgical, trauma)	Academic	Teaching	July 2012
Foothills Medical Centre CVICU	Calgary	Cardiovascular surgical	Academic	Teaching	August 2012
University of Alberta Hospital General Systems ICU	Edmonton	Mixed (medical, surgical, trauma, transplant)	Academic	Teaching	April 2013
University of Alberta Neuro ICU	Edmonton	Neurosciences	Academic	Teaching	June 2013
Mazankowski Alberta Heart Institute CVICU	Edmonton	Cardiovascular surgical	Academic	Teaching	October 2013
Peter Lougheed Hospital ICU	Calgary	Mixed (medical, surgical, vascular)	Tertiary	Teaching	August 2012
Royal Alexandra Hospital ICU	Edmonton	Mixed (medical, surgical, trauma)	Tertiary	Teaching	July 2013
Rockyview General Hospital ICU	Calgary	Mixed (medical, surgical)	Community	Teaching	June 2012
South Health Campus ICU	Calgary	Mixed (medical, surgical)	Community	Community large	February 2013
Sturgeon Community Hospital ICU	St. Albert	Mixed (medical, surgical)	Community	Community large	January 2014
Grey Nuns Community Hospital ICU	Edmonton	Mixed (medical, surgical, vascular)	Community	Teaching	February 2014
Misericordia Community Hospital	Edmonton	Mixed (medical, surgical)	Community	Teaching	March 2014
Medicine Hat Regional Hospital ICU	Medicine Hat	Mixed (medical, surgical)	Regional	Community large	June 2015
Northern Lights Health Centre ICU	Fort McMurray	Mixed (medical, surgical)	Regional	Community medium	November 2015
Chinook Regional Hospital ICU	Lethbridge	Mixed (medical, surgical)	Regional	Community large	December 2015
Grande Prairie QEII Regional Hospital	Grande Prairie	Mixed (medical, surgical)	Regional	Community large	February 2016
Red Deer Regional Hospital ICU	Red Deer	Mixed (medical, surgical)	Regional	Community large	March 2016

Abbreviations: ICU = intensive care unit; CIHI = Canadian Institute for Health Information.

*Hospitals were categorized by Canadian Institute of Health Information by hospital type as follows: teaching (full membership in the Association of Canadian Academic Healthcare Organizations; any size), large (≥200 beds), medium (50-199 beds), and small (1-49 beds) community hospitals.

©2020 Bagshaw SM et al. *JAMA Network Open*.

eTable 2. Summary of Cost-Saving Analysis

<u>Cost saving analysis: potential cost-savings, adjusted for marginal cost.</u>

- Discharged home: n=1,658 (8.3%) ~ total of 2848.2 days avoidable time
- Transferred to ward: n= 18,306 (91.7%) ~ total of 16,525.7 days avoidable time
- Average cost per ICU day: $3,545
- Average cost per ward day: $2,079

Potential savings = (2848.2 days * $3,545/day) + (16,525.7 days * ($3,545-$2,079))

= $10.1 million + $24.2 million= $34.3 million

©2020 Bagshaw SM et al. *JAMA Network Open*.

eTable 3. Avoidable Time in Days and as a Percentage of Total ICU Days by Year

		Discharged		
**Year**	**Avoidable time**	**Ward**	**Home**	**Total**
2012	# days	609	80	688
	% of total ICU days	10.2%	17.3%	10.7%
2013	# days	1,700	223	1,923
	% of total ICU days	7.9%	14.4%	8.3%
2014	# days	4,402	714	5,116
	% of total ICU days	12.3%	18.5%	12.9%
2015	# days	4,260	730	4,990
	% of total ICU days	12.2%	21.1%	13.0%
2016	# days	5,555	1,102	6,657
	% of total ICU days	14.5%	22.2%	15.4%
All years	# days	16,526	2,848	19,374
	% of total ICU days	12.1%	19.9%	12.8%

©2020 Bagshaw SM et al. *JAMA Network Open*.

eTable 4. Costs of Avoidable Time in and as a Percentage of Total ICU Costs by Year

		Discharged		
**Year**	**Cost**	**Ward**	**Home**	**Total**
2012	$ million	$ 0.89	$ 0.28	$ 1.17
	% of total ICU cost	4.2%	17.3%	5.1%
2013	$ million	$ 2.49	$ 0.79	$ 3.28
	% of total ICU cost	3.3%	14.4%	4.0%
2014	$ million	$ 6.45	$ 2.53	$ 8.98
	% of total ICU cost	5.1%	18.5%	6.4%
2015	$ million	$ 6.24	$ 2.59	$ 8.83
	% of total ICU cost	5.1%	21.1%	6.5%
2016	$ million	$ 8.14	$ 3.91	$ 12.05
	% of total ICU cost	6.0%	22.2%	7.8%
All years	$ million	$ 24.23	$ 10.10	$ 34.32
	% of total ICU cost	5.0%	19.9%	6.4%

©2020 Bagshaw SM et al. *JAMA Network Open*.

eTable 5. Sensitivity Analysis of Increasing Home Cost Relative Ward Discharge Cost (per Day) on Total Health Care Costs Attributed to Avoidable Days

Home discharge cost as a proportion of ward discharge cost	Potential cost-savings ($) (all years)			Proportion of total ICU health care costs (%)
	Ward discharge ($)	Home discharge ($)	Total ($)	
Base (0%)	24,226,690.86	10,096,833.55	34,323,524.41	6.4
25%	24,226,690.86	8,616,486.80	32,843,177.66	6.1
50%	24,226,690.86	7,136,140.05	31,362,830.91	5.9
100%	24,226,690.86	4,175,446.54	28,402,137.40	4.4

* Ward cost per day is $2079.

©2020 Bagshaw SM et al. *JAMA Network Open*.

eTable 6. Sensitivity Analysis of the Impact of Potential Reductions in Avoidable Time on Health Care Cost Savings per Year and Potential Added ICU-Bed Capacity That Would Be Available

Potential Reduction in Avoidable-time (% of days)	Potential Cost Savings per Year (CAD $)*	Potential Added ICU-bed Capacity Equivalent per year (No.)
5	$602,552	0.47
10	$1,205,104	0.93
15	$1,807,655	1.40
20	$2,410,207	1.86
25	$3,012,759	2.33
50	$6,025,518	4.66

Note: cost per day for ICU bed $3545

* Based on the total costs of avoidable-time during 2016 (CAD $12,051,036).

©2020 Bagshaw SM et al. *JAMA Network Open*.

eTable 7. Mixed-Effects Cox Regression Model for Association Between In-Hospital Mortality and Having Avoidable-Time

	Univariable analysis	Multivariable analysis
Variable	HR (95% CI)	HR (95% CI)
Avoidable-time	1.0	1.0
Any present	0.70 (0.60-0.81)	0.74 (0.64-0.85)
Sex		
Female	1.0	1.0
Male	0.98 (0.86-1.11)	1.04 (0.91-1.18)
Age		
18-64	1.0	1.0
65-74	2.12 (1.81-2.49)	1.69 (1.43-2.00)
75-84	3.22 (2.74-3.78)	2.54 (2.13-3.02)
≥85	5.65 (4.55-7.02)	4.86 (3.85-6.13)
Patient comorbidity		
Heart failure	1.97 (1.71-2.26)	1.23 (1.06-1.43)
Peripheral vascular disease	1.80 (1.48-2.18)	1.53 (1.25-1.86)
Cerebrovascular disease	1.92 (1.64-2.26)	1.77 (1.49-2.11)
Diabetes mellitus	1.41 (1.24-1.61)	1.09 (0.95-1.25)
Cancer	1.50 (1.28-1.75)	1.37 (1.15-1.63)
Metastatic solid tumor	1.87 (1.53-2.28)	2.10 (1.68-2.62)
Severe/moderate liver disease	2.13 (1.73-2.62)	2.29 (1.76-2.97)
Mild liver disease	1.63 (1.34-2.00)	1.35 (1.06-1.72)
Peptic ulcer	1.48 (1.18-1.86)	1.09 (0.86-1.38)
Rheumatic disease	1.44 (1.06-1.95)	1.21 (0.88-1.65)
Dementia	2.12 (1.66-2.72)	1.31 (0.00-1.69)
Primary diagnostic system		
Cardiovascular	1.0	1.0
Gastrointestinal	0.96 (0.78-1.17)	1.05 (0.84-1.31)
Genitourinary	0.50 (0.32-0.77)	0.64 (0.41-1.00)
Hematology	1.05 (0.39-2.84)	1.68 (0.62-4.56)
Metabolic/endocrine	0.66 (0.36-1.21)	0.78 (0.42-1.44)
Musculoskeletal/skin	0.49 (0.34-0.72)	0.76 (0.52-1.12)
Neurologic	0.46 (0.36-0.59)	0.55 (-.41-0.74)
Respiratory	0.85 (0.69-1.03)	1.04 (0.86-1.27)
Transplant	0.29 (0.13-0.66)	0.42 (0.18-0.98)
Trauma	0.31 (0.23-0.42)	0.88 (0.59-1.31)
Others	0.14 (0.04-0.58)	0.27 (0.07-1.10)
Admission ICU category		
Medical	1.0	1.0
Neurological	0.89 (0.71-1.13)	1.70 (1.25-2.31)
Surgical	0.71 (0.61-0.83)	0.99 (0.75-1.29)
Trauma	0.30 (0.22-0.41)	0.70 (0.44-1.12)
Surgery		

©2020 Bagshaw SM et al. *JAMA Network Open*.

Non-operative	1.0	1.0
Elective	0.42 (0.32-0.54)	0.45 (0.32--.64)
Emergency	0.81 (0.69-0.94)	0.90 (0.70-1.17)
Admission APACHE II score	1.07 (1.06-1.08)	1.05 (1.04-1.06)
Tertiary hospital	0.98 (0.82-1.17)	0.88 (0.74-1.05)
Admission year	0.68 (0.65-0.72)	0.70 (0.66-0.74)

*(yes/no) (n=25,375). Of 28,904 patients, 3,529 patients were excluded for invalid time to event (missing date of death, negative time to event, or ended on or before enter).

©2020 Bagshaw SM et al. *JAMA Network Open*.

eTable 8. Strata of Avoidable-Time and Association With In-Hospital Mortality

Avoidable-time (days)	Hazard Ratio	95% CI	P
0	Reference	-	-
1	0.78	0.67-0.91	0.001
2	0.69	0.55-0.85	0.001
3	0.57	0.42-0.79	0.001
4	0.64	0.42-0.97	0.036
≥5	0.72	0.50-1.02	0.066
**Avoidable-time (days)**	**Hazard Ratio**	**95% CI**	**P**
0	Reference	-	-
1	0.78	0.67-0.91	0.001
2-3	0.65	0.54-0.80	0.000
≥4	0.68	0.51-0.91	0.009
**Avoidable-time (days)**	**Hazard Ratio**	**95% CI**	**P**
0	Reference	-	-
1-3	0.74	0.64-0.86	0.000
≥4	0.69	0.52-0.92	0.010

Cox regression was used to examine risk-adjusted association between in-hospital mortality and avoidable-days stratified by avoidable-time duration prior to ICU discharge. A multilevel mixed effects regression was used to account for variation between ICUs. Primary covariates included patient sex, age, year of admission and whether a patient had avoidable-time at ICU discharge. Likelihood ratio (LR) test was used to examine inclusion of potential risk adjustment factors, including admission APACHE II score and bed occupancy at admission (as continuous), comorbidities and admission time (as binary). Other factors included patient socioeconomic status (by means of median household income at FSA level), primary diagnostic system, admission ICU category, surgical status, and hospital type. Except for the primary variables, a variable remained in the final model if the LR test is significant at a 5% level.

©2020 Bagshaw SM et al. *JAMA Network Open*.

eTable 9. Summary of Duration of Stay Before ICU, Before Discharge Ready in ICU, and Total Lengths of Stay Stratified by Patients Who Did and Did Not Experience Avoidable Time

**Variable**	**Avoidable-time (n=19,964)**	**No avoidable-time (n=8,940)**	**Mean difference (95% CI)**
Pre-ICU stay (d)			
Mean (SD)	2.59 (9.62)	2.39 (8.74)	-0.21 (-0.44 to 0.02)
Median (IQR)	0 (0-2)	0 (0-1)	
Pre-Discharge Ready (hr)			
Mean (SD)	115 (186)	83 (138)	-31.9 (-36.3 to -27.7)
Median (IQR)	57.1 (23-3-126.0)	39.4 (19.3-88.4)	
Post-ICU stay (d)			
Mean (SD)	16.9 (35.7)	10.3 (24.9)	-6.6 (-7.4 to -5.7)
Median (IQR)	7 (3-16)	5 (3-9)	
Total length of stay (d)			
Mean (SD)	25.4 (41.6)	16.4 (29.2)	-9.0 (-9.9 to -8.0)
Median (IQR)	13 (7-26)	9 (5-17)	
Multivariable Analysis[a]	IRR		95% CI
Post-ICU stay	1.27		1.23-1.31
Total stay	1.26		1.23-1.29

[a] adjusted for age, sex, comorbidity, APACHE II score, primary diagnostic classification, surgical status, year, site.

©2020 Bagshaw SM et al. *JAMA Network Open*.

eFigure 1. Summary of Distribution of Avoidable-Time Across Study ICUs During the Study Period

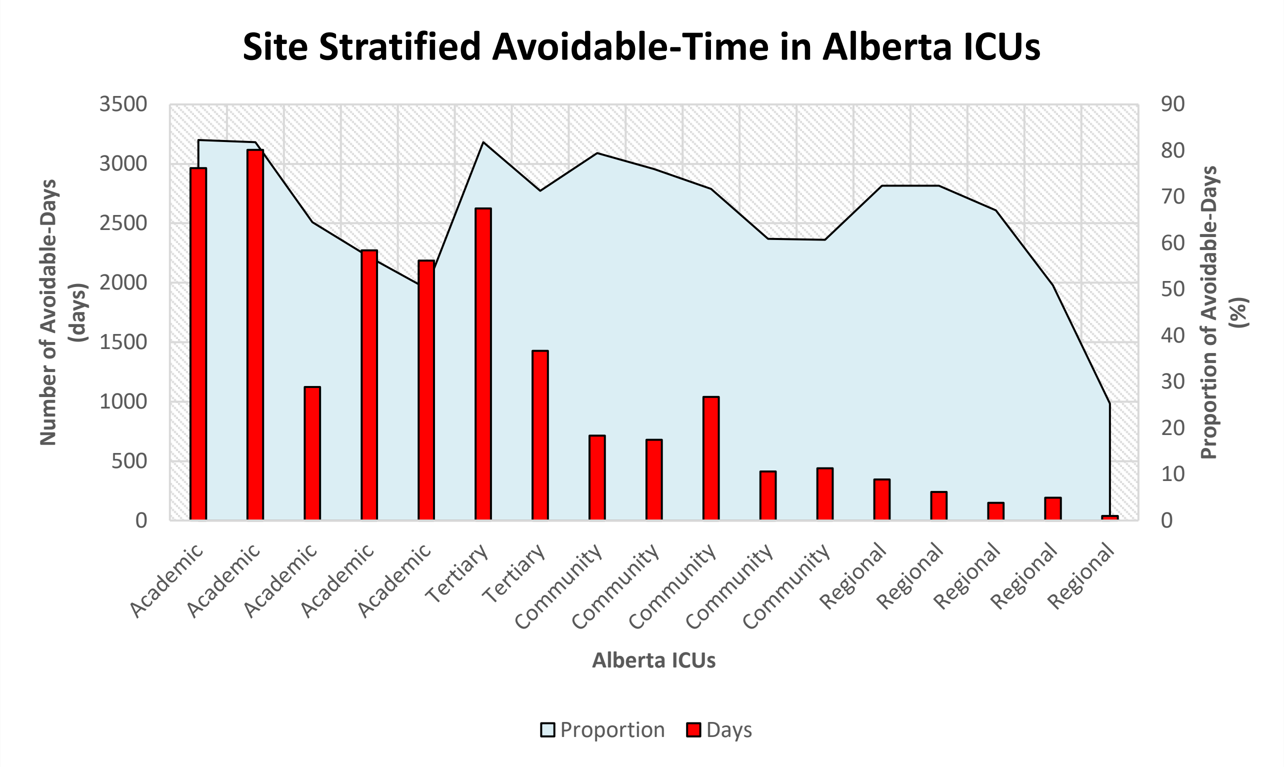

©2020 Bagshaw SM et al. *JAMA Network Open*.

eFigure 2. Summary of ICU Discharge Time Stratified by Avoidable-Time

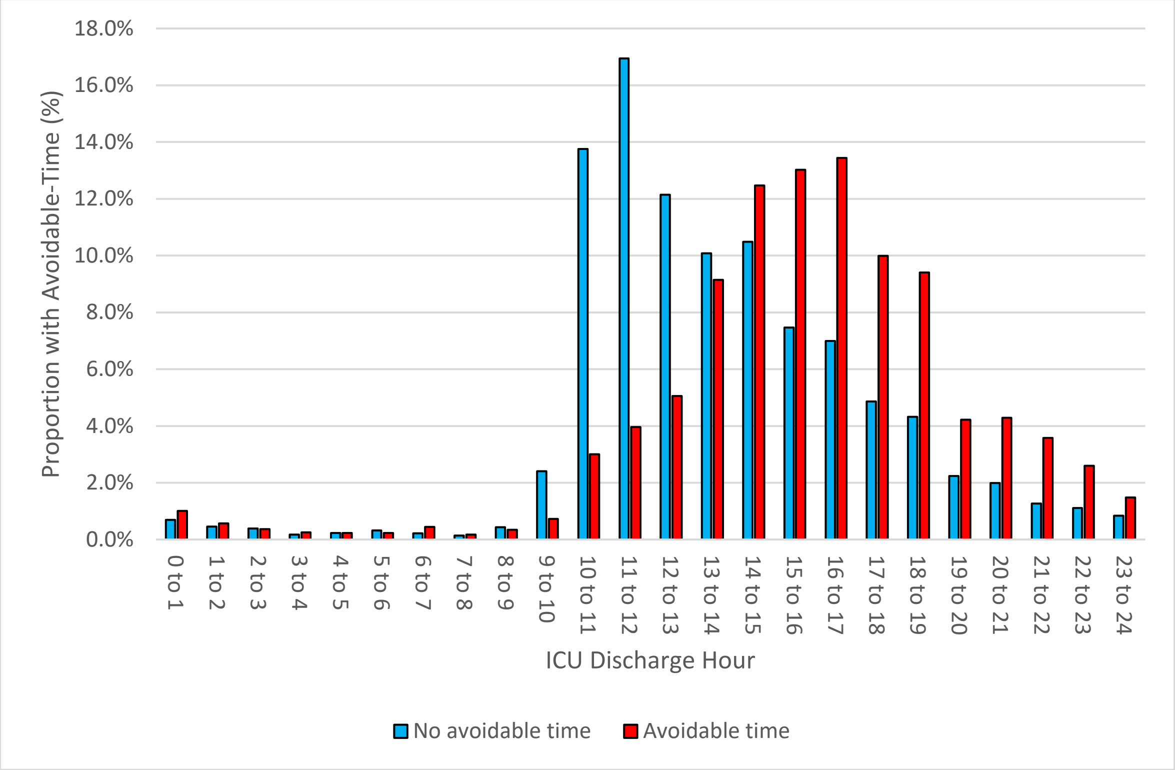

©2020 Bagshaw SM et al. *JAMA Network Open*.
